# Special Issue: Air Pollutant Exposure and Respiratory Diseases

**DOI:** 10.3390/toxics13060441

**Published:** 2025-05-27

**Authors:** Miao He, Wenjun Ding, Kefang Lai

**Affiliations:** 1 Department of Environmental Physical Factors and Health, School of Public Health, China Medical University, Shenyang 110122, China; 2 Laboratory of Environment and Health, College of Life Sciences, University of Chinese Academy of Sciences, Beijing 100049, China; 3 State Key Laboratory of Respiratory Diseases, Guangzhou Institute of Respiratory Health, The First Affiliated Hospital of Guangzhou Medical University, Guangzhou 510120, China

## 1. Introduction

Significant progress has been made in recent research on air pollutant exposure and respiratory diseases. Studies have established a clear association between air pollution and chronic respiratory diseases, with increased pollutant exposure linked to higher disease prevalence and lung function decline [1]. Air pollutants such as fine particulate matter (PM_2.5_) and nitrogen dioxide (NO_2_) can reach deep into the lungs and even enter the bloodstream, causing inflammation and exacerbating chronic respiratory conditions. Proximity to high-pollution sources like major traffic routes is a significant risk factor for these conditions. For instance, long-term exposure to traffic-related air pollution has been shown to increase the risk of asthma and chronic obstructive pulmonary disease (COPD) [2]. PM_2.5_ exposure during childhood also impairs lung development, leading to reduced lung function that can persist into adulthood [3]. Research has also reaffirmed that major air pollutants like PM_2.5_, ozone (O_3_), and nitrogen oxide (NO) can exacerbate the probability of asthma, COPD, respiratory infection, and lung cancer, with the greatest effects often due to particulate matter [4].

Air pollution is an important risk factor enhancing pulmonary disease and causing greater harm in susceptible populations such as children, the elderly and socioeconomically disadvantaged groups [5,6,7]. Even short-term exposures to air pollutants can significantly increase the risk of COPD acute exacerbations [8]. Notably, COPD patients residing in areas compliant with U.S. Environmental Protection Agency (EPA)-national ambient air quality standards (NAAQS) remain at elevated risk of exacerbations following short-term exposures to increased concentrations of sulfur dioxide (SO_2_) [9]. The combined effects of multiple pollutants were also emphasized, such as O_3_ and PM_2.5_ working together to increase respiratory mortality [10].

In summary, although epidemiology has identified adverse effects of ambient air pollution on respiratory health, the pathophysiologic mechanisms are not fully appreciated. This Special Issue focuses on the investigations into pathophysiologic mechanisms underlying the adverse effects of ambient air pollution.

## 2. Overview of the Published Articles

Tert-butylhydroquinone (tBHQ) alleviates particulate matter-induced pyroptosis in human nasal epithelial cells (contribution 1). In Kim et al.’s research, following the exposure of human nasal RPMI 2650 cells to air pollution particles (PM, < 4 µm), NOD-like receptor family pyrin domain containing 3 (NLRP3) inflammasome-dependent pyroptosis is activated through the nuclear factor erythroid 2-related factor 2 (Nrf2) pathway in cells. tBHQ administration effectively inhibits the activation of NLRP3 inflammasome and reduces PM-induced pyroptosis in cells, suggesting that tBHQ may serve as a potential therapeutic agent for PM-related respiratory diseases.

Insecticide exposure contributes to childhood asthma and wheezing (contribution 2). Hu et al. demonstrate the adverse effect of insecticide exposure on childhood respiratory health in Sanya, China. Insecticide exposure is shown to increase the prevalence rates of asthma and wheezing in children. In particular, the adverse impacts are more pronounced in girls who are exposed to low temperatures. This finding underscores the importance of implementing targeted interventions to reduce the health effects of insecticides, particularly in children.

The prevalence of COPD and lung cancer can be affected by ambient air pollutants (contribution 3). Sapbamrer et al. evaluate the relationship between seasonal variation in air pollution and chronic respiratory diseases in Thailand. During the annual haze period, COPD mortality and readmission rates are significantly increased, which is correlated with the elevated levels of PM_10_, PM_2.5_, O_3_, and NO_2_.

An influence of the mitogen-activated protein kinase 1 (MAPK1) pathway on cell apoptosis after PM_2.5_ exposure is characterized by Hong et al. (contribution 4). The PM_2.5_-induced expression of apoptosis-related hsa_circ_0002854 is shown to interact with the MAPK1 protein in 16HBE cells. Changes in biological effect associated with varying hsa_circ_0002854 expression are related to alterations in apoptosis.

Deng et al. define the combined effects of PM_2.5_ and O_3_ on respiratory mortality (contribution 5). The analytical results of a generalized additive regression model (GAM) show that combined exposure to PM_2.5_ and O_3_ significantly increases respiratory mortality, particularly in older adults with lower educational attainment. The health consequence of multiple airborne pollutants are addressed.

Rayanaet et al. apply the job exposure matrix (JEM) approach to retrospectively assess occupational long-term exposure to PM_10_ for a Parisian subway (contribution 6). Annual PM_10_ concentrations are estimated according to the job assignment site over the period of 2004–2020. The difference over time in exposure estimates between locomotive operators’ lines is higher than those between station agents’ sectors. The validated estimates of exposure can be applied in an epidemiological study to investigate the potential health effects of subway PM_10_.

## 3. Conclusions

The six contributions collectively elucidate the multifaceted impacts of environmental pollutants on respiratory health. They cover various aspects, including the potential therapeutic role of antioxidants in mitigating PM-induced inflammation and cell death, the link between insecticide exposure and childhood respiratory health, long-term trends in air pollutants and their association with chronic respiratory diseases, the molecular mechanisms of PM_2.5_-induced respiratory diseases, the combined effects of O_3_ and PM_2.5_ on respiratory mortality, and the use of a job exposure matrix for the retrospective assessment of particle exposure in subway systems. These studies enhance our understanding of the respiratory effects of environmental pollutants and highlight the potential avenues for intervention and mitigation strategies.

## References

[1] Doiron D., de Hoogh K., Probst-Hensch N., Fortier I., Cai Y., De Matteis S., Hansell A.L. (2019). Air pollution, lung function and COPD: Results from the population-based UK Biobank study. Eur. Respir. J..

[2] Lindgren A., Stroh E., Montnemery P., Nihlen U., Jakobsson K., Axmon A. (2009). Traffic-related air pollution associated with prevalence of asthma and COPD/chronic bronchitis. A cross-sectional study in Southern Sweden. Int. J. Health Geogr..

[3] Dubrowski A., Klis K., Zurawiecka M., Deren K., Barszcz M., Nowakowski D., Wronka I. (2019). Long-Term Exposure to Ambient Air Pollution in Childhood-Adolescence and Lung Function in Adulthood. Adv. Exp. Med. Biol..

[4] Kelly F.J., Fussell J.C. (2011). Air pollution and airway disease. Clin. Exp. Allergy.

[5] Meme H., Amukoye E., Bowyer C., Chakaya J., Das D., Dobson R., Dragosits U., Fuld J., Gray C., Hahn M. (2023). Asthma symptoms, spirometry and air pollution exposure in schoolchildren in an informal settlement and an affluent area of Nairobi, Kenya. Thorax.

[6] Wang M., Aaron C.P., Madrigano J., Hoffman E.A., Angelini E., Yang J., Laine A., Vetterli T.M., Kinney P.L., Sampson P.D. (2019). Association Between Long-term Exposure to Ambient Air Pollution and Change in Quantitatively Assessed Emphysema and Lung Function. JAMA.

[7] Jabre N.A., Keet C.A., McCormack M., Peng R., Balcer-Whaley S., Matsui E.C. (2020). Material Hardship and Indoor Allergen Exposure among Low-Income, Urban, Minority Children with Persistent Asthma. J. Community Health.

[8] de Rooij M.M.T., Erbrink H.J., Smit L.A.M., Wouters I.M., Hoek G., Heederik D.J.J. (2024). Short-term residential exposure to endotoxin emitted from livestock farms in relation to lung function in non-farming residents. Environ. Res..

[9] DeVries R., Kriebel D., Sama S. (2016). Low level air pollution and exacerbation of existing copd: A case crossover analysis. Environ. Health.

[10] Liu C., Chen R., Sera F., Vicedo-Cabrera A.M., Guo Y., Tong S., Lavigne E., Correa P.M., Ortega N.V., Achilleos S. (2023). Interactive effects of ambient fine particulate matter and ozone on daily mortality in 372 cities: Two stage time series analysis. BMJ.

